# Sleep disturbance and psychological distress are associated with functional dyspepsia based on Rome III criteria

**DOI:** 10.1186/s12888-018-1720-0

**Published:** 2018-05-18

**Authors:** Yong Li, Yaoyao Gong, Yinghui Li, Danjun He, Yuqin Wu, Haofei Wang, Xiaoyin Cong, Muxin Wei, Lin Lin

**Affiliations:** 1 Department of Psychiatry, the First Affiliated Hospital of Nanjing Medical University, Nanjing, 210029 China; 2 Department of Gastroenterology, the First Affiliated Hospital of Nanjing Medical University, Nanjing, 210029 China; 3 Department of Traditional Chinese Medicine, the First Affiliated Hospital of Nanjing Medical University, Nanjing, 210029 China; 4 Department of Psychiatry, Zhongda Hospital, Southeast University, Nanjing, 210029 China

**Keywords:** Functional dyspepsia, Sleep disturbance, Psychological factors, Rome III criteria

## Abstract

**Background:**

Functional dyspepsia (FD) is considered a bio-psychosocial disorder. The role of psychosocial factors in FD pathogenesis remains unclear.

**Methods:**

This study evaluated sleep quality and mood symptoms in patients with FD, assessing the associations of FD severity, disordered sleep, and psychological symptoms. One-hundred-and-fifteen adult patients with typical FD symptoms were enrolled alongside 61 healthy volunteers. Rome III criteria were used to evaluate FD symptoms; sleep disorder was assessed with the Pittsburgh Sleep Quality Index (PSQI), and Symptom Checklist-90-Revised (SCL-90R) was utilized to determine the status of depression, anxiety and other psychological symptoms.

**Results:**

PSQI scores and nine symptomatic dimensions of SCL-90R were significantly higher in FD patients than in controls. Multiple logistic regression indicated that lower BMI, lower level of education, and sleep disturbance were independently associated with FD and FD subgroups. Hostility and phobic anxiety were independent risk factors for FD. Further analysis showed that hostility was an independent risk factor for both FD subgroups, and somatization and additional psychiatric symptoms for epigastric pain syndrome.

**Conclusions:**

We found that FD was associated with sleep disorder and psychopathological factors. These findings suggest that implementing sleeping and/or psychological therapies may help reduce FD symptoms.

## Background

Functional dyspepsia (FD), one of the most common functional gastrointestinal disorders (FGIDs), is defined as pain and/or discomfort centered in the upper abdomen in the absence of obvious organic abnormalities [1]. The Rome III Consensus divided FD into two subgroups: epigastric pain syndrome (EPS, characterized by epigastric pain and burning) and postprandial distress syndrome (PDS, characterized by postprandial fullness and early satiety). FD is currently considered a bio-psychosocial disorder as a result of impaired motor function, *Helicobacter pylori* infection, heightened visceral sensitivity, central nervous system disturbance, and unhealthy lifestyle [2]. Due to the differences in dietary habits, lifestyle, and *Helicobacter pylori* infection prevalence, the symptoms, epidemiology, pathogenetic factors, and management differ between Asian and Western FD patients [3].

Mounting evidence suggests that psychological distress is associated with FD; however, very few studies have assessed psychological factors that contribute to FD development. A Swedish 10-year-follow-up study of FD patients applying the Rome III definition showed an association of anxiety but not depression with FD [4]; meanwhile, a retrospective study based on the Rome III definition revealed that postprandial distress syndrome but not epigastric pain syndrome is associated with somatization, depression, and phobia [5].

Besides psychological factors, sleep disorder is a common phenomenon in FD patients and may influence the severity of dyspepsia symptoms [6]. In addition, sleep disturbance, anxiety, and depression are usually closely related; previous studies also found that sleep disturbance in FD patients appears to be associated with higher levels of anxiety [6]. However, whether sleep disorder or various psychological factors are independently associated with the development and severity of FD remains unclear.

In the present study, we used an integrated questionnaire consisting of the major dyspepsia symptom scale, Symptom Checklist-90-Revised (SCL-90R), and Pittsburgh Sleep Quality Index (PSQI) to assess FD severity, psychological health and sleeping condition in patients meeting Rome III criteria for FD. The study aimed to explore the associations of psychological distress or sleep disturbance with FD and FD subgroups based on Rome III criteria.

## Methods

### Participants and setting

Adult outpatients from the digestive and psychological departments of First Affiliated Hospital of Nanjing Medical University meeting ROME III criteria for FD between July 2015 and May 2016 were recruited for this study. The included patients have undergone an upper endoscopic test to exclude organic digestive diseases that might have explained the observed symptoms. Local adult volunteers without known gastrointestinal or psychological disorders, who underwent gastric endoscopy as regular medical examination, were recruited as healthy controls. All patients and controls underwent detailed history taking. All subjects with the following conditions were excluded: (a) a history of upper gastrointestinal tract surgery, (b) a history of ulcerative disease or malignancy, (c) current treatment with non-steroidal anti-inflammatory drugs (NSAIDs), (d) use of monoamine oxidase inhibitors or selective serotonin reuptake inhibitors, (e) pregnancy for women, (f) pancreatitis or hepatobiliary disorders, and (g) physical or psychological impairments that prevented from completing the questionnaires. Patients with concomitant irritable bowel syndrome (IBS) symptoms but not meeting the ROME III criteria for IBS were not excluded.

### FD symptoms definition and Rome III diagnostic questionnare

FD was defined as the presence of postprandial fullness/early satiation or pain/burning more than once per week, in the last 3 months according to the Rome III criteria. To diagnose FD and evaluate the severity of dyspepsia related symptoms, the original Rome III functional dyspepsia module [1] was translated into Chinese, confirmed by two digestive disease experts, and scrutinized by two native Chinese speakers without medical background to ensure that the text was easily understandable. The severity of major dyspeptic symptoms (postprandial abdominal fullness or discomfort, early satiety, epigastric pain, and epigastric burning) was rated on a 7-point Likert scale (0, absent; 1, hardly any; 2, mild; 3, moderate; 4, moderately severe; 5, severe; 6, very severe).

### Sleep quality assessment (PSQI)

A Chinese version of the PSQI questionnaire was used to assess the patient’s recent history of sleep quality. The PSQI questionnaire consisted of 19 self-rating items that can be categorized into seven components, including subjective sleep quality, sleep latency, sleep duration, habitual sleep efficiency, sleep disturbance, use of sleep medication, and daytime dysfunction. The score of each component score was rated from 0 to 3. The sum of these seven component scores provided a global PSQI score, which ranged from 0 to 21. Higher scores indicate poorer sleep. The Chinese Version of PSQI was translated and validated by Liu et al. [7]. In that study, the internal consistency Cronbach’s α was 0.84, the split-half reliability was 0.87, and the 2-week test-retest reliability was 0.81.

### Evaluation of psychiatric distress and lifestyle factors

Psychiatric distress was estimated using the Chinese version of SCL-90R [8]. The SCL-90R is a self-reporting, clinical symptom rating scale consisting of 90 items. Responses indicate symptoms associated with 9 symptom dimensions, which included somatization (distress arising from perceptions of bodily dysfunction), obsessive-compulsive (thoughts, impulses and actions that are experienced as irresistible and unremitting), interpersonal sensitivity (feelings of inferiority or inadequacy), depression, anxiety, hostility (thoughts, feelings and actions that are characteristic of the negative affect state of anger), phobic anxiety (irrational fear of specific situations or persons), paranoid ideation (paranoid behavior as fundamentally a disordered mode of thinking), and psychoticism [9]. Additionally, a global symptom score is calculated. The Chinese-version SCL-90R questionnaire [8] with established reliability and validity is widely used in measuring psychological distress in Chinese clinical practice and research. In a recent study carried out in patients with alopecia areata, the internal consistency Cronbach’s α was 0.98 and split-half coefficient was 0.95 [10]. Demographic data and lifestyle factors (including age, gender, marital status, body mass index (BMI), education level, smoking, and consumption of alcohol were recorded in all patients and controls). Tobacco and alcohol use was divided into never/rare and regular use (more than once a week). Each questionnaire was completed individually and independently by the participant.

### Data analysis

Data were assessed with Statistical Package for the Social Sciences (SPSS) for Windows, version 13.0. Measurement data are mean ± standard deviation (SD). Student *t* test and one way analysis of variance (ANOVA) were used to statistically assess demographic variables such as weight, height, and BMI. Various sleep disorder parameters and psychological factors between FD patients and healthy controls were also assessed by Student *t* test. The association of total PSQI score and FD symptom severity score was assessed by Spearman correlation analysis. Risk factors for dyspepsia were initially explored by univariate analysis. Those with *P* < 0.05 were then entered into a multivariate analysis. Independent risk factors for dyspepsia were subsequently determined by backward elimination logistic regression. Data were expressed as odds ratio (OR) with 95% confidence interval (95% CI). *P* < 0.05 was considered statistically significant.

## Results

### Response rate and demographics

Of the 263 subjects eligible for inclusion, a total of 224 (85.1%) individuals agreed to participate in the study. However, 109 individuals did not complete the questionnaires because of low education level or desire to withdraw. Therefore, a total of 115 FD outpatients completed questionnaires, yielding a response rate of 43.7%. Meanwhile, 61 healthy controls were enrolled. The mean age of the control population was 41.36 ± 12.32 years; 80.3% of control individuals were female.

Table 1 describes the basic demography of the study population. The mean age of interviewees was 41.72 ± 12.22 years, ranging from 17 to 61 years. A total of 143 (81.3%) adults were female. There were no significant differences between the two groups in terms of age, gender, marital status, regular tobacco use, and regular alcohol intake. FD patients had lower BMI and level of education compared with the control group.

**Table 1:** Basic demographics of the study population

Characteristic	FD (*n* = 115)	Controls (*n* = 61)	*P* value
Age (year)	41.90 ± 12.21	41.36 ± 12.32	0.78
BMI	23.46 ± 2.22	24.99 ± 2.40	0.00*
Gender Female (%)	94 (81.7)	49 (80.3)	0.82
Marital status			
Single/widower/divorced (%)	21 (18.5)	6 (10.0)	0.14
Married (%)	94 (81.7)	54 (90.0)	0.15
Years of education (year)	11.31 ± 2.81	13.95 ± 4.04	0.00*
Regular tobacco use (%)	42 (36.5)	22 (36.7)	0.98
Regular alcohol intake (%)	44 (38.3)	25 (41.7)	0.66
Duration of symptoms (mo)	52 ± 50	–	–
Days per week with symptoms	4.1 ± 2.3	–	–

*BMI* body mass index calculated as weight (kg)/height (m^2^)

*P < 0.05

### FD symptoms

Mean duration of FD symptoms was 52 ± 50 months, and the patients reported that FD symptoms were present 4.1 ± 2.3 days per week. Among the 115 FD participants, 87 (75.7%) had symptoms consistent with EPS (including 61 individuals with pure EPS symptoms) and 54 (47.0%) conformed to PDS (including 28 with pure PDS symptoms); 26 patients (22.6%) reported dyspeptic symptoms compatible with both EPS and PDS.

### Sleep symptoms

Average sleep durations in FD patients and healthy controls were 6.24 ± 1.37 and 6.87 ± 1.05 h, respectively (*P* < 0.05). Occasional sleep problems were common in both FD and control groups; indeed, 67.2 and 58.6% of FD patients and control participants, respectively, reported having sleep abnormality 1 to 3 days a month. Significantly higher PSQI scores were found in FD patients compared with controls (10.49 ± 3.38 vs 6.77 ± 2.77, respectively; *P* < 0.01). In addition, as shown in Table 2, six parameters, including subjective sleep quality, sleep latency, sleep duration, sleep disturbance, use of sleeping medication, and daytime dysfunction showed remarkably higher scores in FD patients compared with controls.

**Table 2:** PSQI scores in FD patients and healthy controls (mean ± SD)

Groups	Subjective sleep quality	Sleep latency	Sleep duration	Habitual sleep efficiency	Sleep disturbance	Use of sleeping medications	Daytime dysfunction	Total score
FD	1.94 ± 0.61	2.43 ± 0.85	1.40 ± 1.11	1.33 ± 1.39	1.81 ± 0.51	0.70 ± 1.16	0.75 ± 0.66	10.49 ± 3.38
HC	1.61 ± 0.86	1.60 ± 1.13	0.89 ± 0.91	1.08 ± 1.08	1.18 ± 0.89	0.21 ± 0.41	0.21 ± 0.49	6.77 ± 2.77
*t*	2.678	5.065	3.298	1.306	5.110	4.019	6.102	7.830
*P*	0.009*	0.000*	0.001*	0.193	0.000*	0.000*	0.000*	0.000*

*P < 0.05

### Association of Sleep Disturbance with FD symptoms

**Fig. 1:**
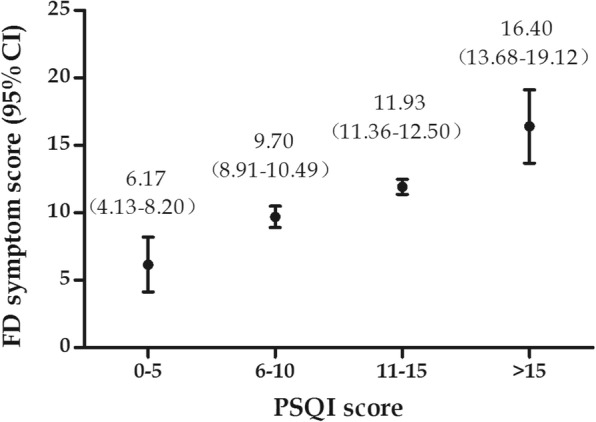
Univariate association of PSQI score with FD symptom score. The FD symptom score increased stepwise with sleep score. The higher the sleep score, the more severe the sleep disturbance was

To further investigate the association between overall sleep score and FD symptom score, we applied a Spearman regression analysis within patients with FD. There was a significant correlation between total PSQI sleep score and overall FD symptom score (*r*_*p*_ = 0.63, *P* < 0.01). A total of 61 FD patients had a score above 11, with 5 having values > 15, which reflects severe sleep disturbance in all domains. Moreover, there was a consistent, stepwise increase of FD symptom scores with increasing sleep scores (Fig. 1).

### Psychological symptoms

To assess the associations of various psychological factors with FD development, we first compared ten symptomatic dimensions as well as total SCL-90R scores between FD patients and healthy controls. Interestingly, somatization, obsessive-compulsive, interpersonal sensitivity, depression, anxiety, hostility, phobic anxiety, psychoticism, additional items, and total score showed markedly higher levels in the FD group than in controls (Table 3).

**Table 3:** SCL-90R scores in FD patients and healthy controls (mean ± SD)

Groups	Somatization	Obsessive-compulsive	Interpersonal sensitivity	Depression	Anxiety	Hostility	Phobic anxiety	Paranoid ideation	Psychoticism	Additional items	Total score
FD	1.75 ± 0.74	2.00 ± 0.77	1.81 ± 0.82	1.87 ± 0.88	1.78 ± 0.83	1.84 ± 0.81	1.53 ± 0.83	1.73 ± 0.82	1.63 ± 0.70	1.90 ± 0.90	162.00 ± 66.26
HC	1.40 ± 0.43	1.75 ± 0.56	1.52 ± 0.43	1.53 ± 0.54	1.47 ± 0.43	1.52 ± 0.61	1.25 ± 0.36	1.55 ± 0.55	1.42 ± 0.40	1.58 ± 0.56	135.18 ± 36.80
*t*	3.960	2.485	3.026	3.146	3.212	3.018	3.155	1.738	2.536	2.911	3.452
*P*	< 0.000*	0.014*	0.003*	0.002*	0.002*	0.003*	0.002*	0.084	0.012*	0.004	0.001*

*P < 0.05

To further identify which psychological factors were independent risk factors for FD, a multivariate logistic regression model was employed (Table 4). Hostility (OR = 4.29, 95%CI 1.12–16.47, *P* < 0.05) and phobic anxiety (OR = 6.41, 95%CI = 1.40–29.43, *P* < 0.05) were independent risk factors for dyspepsia. However, no significant associations of symptomatic dimension scores with the FD symptom score were found.

**Table 4:** Univariate and multivariate logistic regression analysis of risk factors for FD

Variables	Univariate analysis			Multivariate analysis		
	OR	95%CI	*P*	OR	95%CI	*P*
Age	1.004	0.978–1.030	0.778	–	–	–
Gender	1.096	0.498–2.413	0.819	–	–	–
BMI	0.749	0.646–0.868	< 0.001	0.750	0.619–0.910	0.004*
Years of education	0.788	0.709–0.875	< 0.001	0.771	0.669–0.888	< 0.001*
PSQI score	1.451	1.276–1.649	< 0.001	1.549	1.301–1.843	< 0.001*
SCL-90R score	1.009	1.003–1.016	0.006	0.963	0.866–1.070	0.484
Somatization	2.617	1.433–4.779	0.002	4.365	0.603–31.623	0.145
Obsessive-compulsive	1.735	1.063–2.832	0.027	1.260	0.181–8.765	0.815
Interpersonal sensitivity	1.910	1.133–3.219	0.015	3.344	0.374–29.927	0.280
Depression	1.880	1.169–3.024	0.009	4.362	0.347–54.826	0.254
Anxiety	2.041	1.179–3.536	0.011	0.492	0.037–6.477	0.590
Hostility	1.953	1.187–3.212	0.008	4.287	1.116–16.467	0.034*
Phobic anxiety	2.077	1.137–3.794	0.017	6.411	1.396–29.433	0.017*
Paranoid ideation	1.427	0.904–2.252	0.127	–	–	–
Psychoticism	1.865	1.044–3.331	0.035	0.147	0.021–1.004	0.050
Additional items	1.742	1.117–2.719	0.014	0.349	0.094–1.292	0.115

*P < 0.05

### Independent risk factors for FD

Besides hostility and phobic anxiety, lower BMI (OR = 0.75; 95%CI 0.62–0.91; *P* < 0.05), lower education levels (OR = 0.77; 95%CI 0.67–0.89; *P* < 0.001), and higher PSQI score (OR = 1.55; 95%CI 1.30–1.84; *P* < 0.001) were also independent risk factors for FD (Table 4).

### Independent risk factors for FD subgroups

To assess the associations of each FD subgroup with psychiatric distress and sleep disorder, subgroup analysis was performed. EPS was independently associated with somatization, hostility and additional symptoms, while PDS was independently associated with hostility. Both subgroups were independently associated with lower BMI, lower education level, and higher PSQI score (Table 5).

**Table 5:** Multivariate logistic regression analysis of risk factors for FD subgroups

Variables	EPS			PDS		
	OR	95%CI	*P*	OR	95%CI	*P*
BMI	0.768	0.625–0.944	0.012*	0.560	0.406-0.771	< 0.001*
Years of education	0.800	0.691–0.925	0.003*	0.701	0.574-0.857	0.001*
PSQI score	1.531	1.287–1.822	< 0.001*	1.690	1.297-2.201	< 0.001*
SCL-90R score	0.942	0.854–1.038	0.227	0.832	0.578–1.197	0.321
Somatization	8.571	1.096–67.017	0.041*	16.132	0.147-1771.600	0.246
Obsessive-compulsive	1.176	0.171–8.093	0.869	2.566	0.047–139.886	0.644
Interpersonal sensitivity	3.813	0.391–37.185	0.249	50.783	0.755–3415.805	0.067
Depression	2.789	0.212–36.660	0.435	110.215	0.408–29,799.742	0.100
Anxiety	1.709	0.148–19.761	0.668	0.462	0.003–72.625	0.765
Hostility	3.722	1.005–13.792	0.049*	16.826	1.093-259.051	0.043*
Phobic anxiety	3.534	0.906–13.783	0.069	14.084	0.575–344.787	0.105
Paranoid ideation	–	–	–	0.843	0.029–24.241	0.920
Psychoticism	–	–	–	2.939	0.051–170.797	0.603
Additional items	0.241	0.074–0.786	0.018*	0.990	0.066-14.862	0.994

*P < 0.05

## Discussion

Previous studies suggested that psychological distress is common in patients with FD [4, 5, 11, 12]. However, the psychological factors associated with FD remain undefined. In this study, both PSQI and SCL-90R were applied to assess the sleeping and psychological conditions of patients with FD, analyzing the associations of sleep disturbance, somatization, obsessive-compulsive, interpersonal sensitivity, depression, anxiety, hostility, phobic anxiety, paranoid ideation and psychoticism with FD, respectively. We found that FD was associated with sleep disorder and several psychopathological factors (somatization, obsessive-compulsive, interpersonal sensitivity, depression, anxiety, hostility, phobic anxiety, psychoticism). In addition, we found that lower BMI, lower level of education, sleep disorder, hostility and phobic anxiety were independent risk factors for FD.

The associations of poor sleep with functional gastrointestinal disorders have been evaluated in previous physiological and epidemiological studies [6, 13–17]. However, whether sleep disturbance is independently associated with FD remains controversial. A previous study assessing 505 patients with functional gastrointestinal disorders and 247 healthy controls found that 68 and 50.2% of individuals with FD and irritable bowel syndrome (IBS), respectively, have sleep disturbance [15]. A study based on Rome III criteria for FD showed that FD symptoms are significantly correlated with sleep disturbance [6]. A community-based cohort study reported that sleep dysfunction and somatization are independent risk factors for dyspepsia [14]. A population-based research indicated that IBS is significantly more common in people with sleep disturbance, while its association with FD was not statistically significant [13]. In the present study, sleep disturbance was an independent risk factor for FD and both FD subgroups. In addition, PSQI scores were higher in FD patients with more severe symptoms, suggesting that impaired sleep may be an important factor affecting the severity of FD symptoms.

The mechanisms underlying mutual interactions between sleep disturbance and FD symptom expression may be quite complex. We hypothesized that on one hand, severe epigastric pain or burning may lead to disorders that initiate and maintain sleep; on the other hand, disrupted sleep could lead to increased symptom expression in patients with FD. In support of this hypothesis, a previous study demonstrated that both rapid eye movement (REM) sleep and slow wave sleep (SWS) interruption could decrease mechanical pain thresholds in healthy volunteers [18].

The role of adverse psychological factors in the development of functional gastrointestinal disorders (FGIDs) attracts increasing attention [19]. In a recent retrospective study, data of 4966 patients diagnosed with a FGID and 1002 randomly selected individuals indicated that twice as much patients are diagnosed with a mood disorder before an FGID; mood disorder was on average diagnosed more than 3 years before FGID [20]. As mentioned above, studies assessing the psychological factors that contribute to FD development are limited. In this study, SCL-90R was applied to assess mood condition, and the scores of 9 symptomatic dimensions, including somatization, obsessive-compulsive, interpersonal sensitivity, depression, anxiety, hostility, phobic anxiety, psychoticism and additional items, were markedly higher in patients with FD than in healthy controls. These findings demonstrated that negative emotions are common in patients with FD. Given the chronicity of symptoms in the current patient cohort, this is unsurprising because chronic disease status may cause increased prevalence of mood disorders. Further analysis of independent risk factors for FD indicated that hostility and phobic anxiety were independently associated with FD; subgroup analysis showed that hostility was an independent risk factor for both FD subgroups, while somatization and additional psychiatric symptoms predicted EPS.

With respect to clinical application, whether treating FD patients with sleep or psychotherapeutic medications improves dyspeptic symptoms remains unclear. Related data are limited, as only few randomized controlled trials have been conducted for FD psychotherapy [21, 22]. A systematic review assessing the effects of psychotherapy on dyspepsia symptoms was withdrawn because only four trials were eligible [5]. Nevertheless, all four trials suggested that psychological interventions benefit dyspepsia symptoms [21]. A 6-month-follow-up randomized controlled trial reported that adding psychotherapy to standard medical therapy improves short- and long-term curative effects [22]. In a recent randomized, placebo-controlled trial, treating FD patients with mirtazapine was shown to significantly improve early satiation, the quality of life, gastrointestinal-specific anxiety, nutrient tolerance, and weight loss [23].

This is a cross-disciplinary research spanning gastroenterology and psychology. We focused on evaluating sleep quality and mood symptoms in Chinese patients with FD, with a view to compare with other racial population in future. It is helpful for clinicians to pay more attention to the sleep disturbance and psychological factors of FD patients and to provide more effective treatments for them.

The present study had several limitations. For example, no physiological measurements were used in this study, although the diagnosis of sleep disturbance is still largely dependent on patient’s subjective feelings and symptoms.

## Conclusions

In conclusion, this study demonstrated that sleep disturbance and negative emotions were common in patients with FD. Impaired sleep was an independent risk factor for FD and both FD subgroups, and may play a role in symptom expression. Hostility and phobic anxiety were independently associated with FD development. We further demonstrated that hostility was an independent risk factor for both FD subgroups, while somatization and additional psychiatric symptoms predicted EPS. Further exploration of the mechanisms linking sleep disturbance and negative emotions with FD may yield new approaches to interventions that reduce FD symptoms.

## Abbreviations

BMI: Body mass index
EPS: Epigastric pain syndrome
FD: Functional dyspepsia
FGIDs: Functional gastrointestinal disorders
IBS: Irritable bowel syndrome
PDS: Postprandial distress syndrome
PSQI: Pittsburgh Sleep Quality Index
REM: Rapid eye movement
SCL-90R: Symptom Checklist-90-Revised
SWS: Slow wave sleep
